# The systemic costs of hematopoietic stem cell aging

**DOI:** 10.1242/dev.205103

**Published:** 2025-10-27

**Authors:** Gayatri Puri, Roméo S. Blanc

**Affiliations:** Department of Cell and Regenerative Biology, University of Wisconsin-Madison, Madison, WI 53705, USA

**Keywords:** Systemic aging, Bone marrow, Hematopoietic stem cell, Inflammation, Immunomodulation, Immunosenescence

## Abstract

Stem cell behavior is tightly regulated by signals from the surrounding immune environment. Immune cells play an indispensable role in the maintenance, activation and differentiation of tissue-resident stem cells (TSCs). These interactions are dynamic and adapt across the lifespan, profoundly influencing regenerative capacity under both physiological and pathological conditions. Notably, immune dysfunction originating from aging hematopoietic stem cells (HSCs) disrupts tissue regeneration across distant organs, including the brain, muscle and skin. In this Review, we synthesize current knowledge on the interplay between HSC aging and TSC function, emphasizing how age-related changes in HSC-derived immune outputs impair local tissue homeostasis. We explore potential mechanisms underlying HSC–TSC communication, including inflammaging, cytokine signaling and the secretion of bioactive factors. Finally, we discuss emerging strategies aimed at rejuvenating aged HSCs, restoring immune equilibrium and enhancing systemic tissue regeneration. By linking systemic immune remodeling to local niche dysfunction, this Review proposes a hierarchical model in which HSC aging acts as a central regulator of tissue regenerative decline.

## Teaser

**Summary:** This Review discusses the effects of aging hematopoietic stem cells, highlighting direct and indirect crosstalk within various tissues and potential therapeutic strategies to rejuvenate hematopoiesis and restore systemic repair.

## Introduction

The transition from early adulthood to old age is accompanied by dynamic and progressive changes in tissue homeostasis and regenerative capacity. The local environment within each tissue is central to its maintenance across the lifespan and serves as a niche for tissue-resident stem cells (TSCs). These niches also contain a rich array of immune cells, including macrophages, neutrophils, dendritic cells and lymphocytes (Davies et al., 2013; Fan and Rudensky, 2016). There has been an increasing appreciation for the non-canonical functions of immune cells aside from protection against pathogens. The composition of immune cells (along with their secreted cytokines and chemokines) are tightly regulated but change dramatically with age and physiological transitions (Liu et al., 2023b). Age-dependent transformations within each niche can either support stem cell quiescence, activation and repair, or, conversely, drive dysfunction and exhaustion. It is now widely accepted that local immune remodeling in tissues such as muscle, skin, brain and heart alters the regenerative response of resident stem/progenitor cell in response to stress and injury (Abnave and Ghigo, 2019). Thus, the interplay between the immune system and tissue-specific stem cells may play an important role in healthy aging and resilience to disease.

Although many of these immune changes reflect tissue-intrinsic factors, systemic influences originating from the hematopoietic system also have far-reaching effects on distal niches. Under normal physiological conditions, hematopoietic stem cells (HSCs), residing in the bone marrow (BM) niche, maintain lifelong replenishment of blood and immune cells. Although HSC numbers typically increase with age, their self-renewal capability significantly diminishes (Dykstra and de Haan, 2008). Additionally, aged HSCs exhibit skewed differentiation toward myeloid lineages, producing fewer lymphoid progenitors (B and T cells) and more megakaryocytic cells (Dykstra et al., 2011; Pang et al., 2011; Grover et al., 2016; Aksöz et al., 2024). The notion of erythroid bias in aged HSCs remains controversial, as some studies report expansion of erythroid progenitors while others show their decline. Given the lack of consensus, we have not discussed erythroid output in detail here (Li et al., 2025; Grover et al., 2016). These shifts promote enhanced myelopoiesis, resulting in myeloid populations with reduced phagocytic efficiency and diminished antigen-presenting capacity (Dorshkind et al., 2009). Alongside aging of peripheral immune compartments, such as thymus and spleen, these HSC-associated shifts contribute to disrupted immune balance and impaired inter-tissue communication (Budamagunta et al., 2021; Dorshkind and Swain, 2009). Aging within the BM niche and HSC compartment significantly impacts distal stem cell populations, positioning HSC aging as a key driver of systemic tissue decline. A study showed that DNA methylation signatures derived from blood cells not only estimate biological age but also reflect HSC aging, lineage bias and immune remodeling (Paparazzo et al., 2022). Given that mature blood cells originate from HSCs, their methylation patterns fundamentally mirror the epigenetic and functional status of the hematopoietic system, underscoring the systemic impact of hematopoietic aging.

Moreover, advances in multi-omics, single-cell profiling and computational modeling have revealed that immune remodeling within both local and systemic environments is not a late-stage event but rather a continuum, affecting tissue maintenance and repair from early adulthood onward (Gao et al., 2024; Paparazzo et al., 2022). Understanding how immune system variation within tissue niches orchestrates lifelong stem cell function is therefore crucial for developing interventions to preserve health span and regenerative capacity.

This Review begins by exploring how immune cell composition and function changes within different stem cell niches across the adult lifespan, and how these changes shape the behavior of TSCs. We then discuss emerging evidence implicating hematopoietic aging as a key driver of systemic immune remodeling, followed by mechanisms underlying age-related changes in HSC differentiation and potential strategies to restore immune-stem cell homeostasis.

## Crosstalk between HSC aging and TSC aging

The tissue microenvironment, or niche, is not only structural support for TSCs but also an immunologically active compartment, with immune activity regulating tissue regeneration either directly by affecting stem cells or indirectly via niche-mediated factors. Throughout the lifespan, the composition, abundance and function of immune cells within these niches are subjected to profound and dynamic remodeling, influenced by both intrinsic tissue signals and systemic factors (Kumar et al., 2018). Early in adulthood, immune cells, including macrophages, dendritic cells and tissue-resident memory T cells, contribute to immune surveillance, resolution of inflammation, and (by releasing niche-supportive cytokines and chemokines) tissue repair (Manole et al., 2021). As individuals transition from early adulthood to old age, these immune populations become skewed towards a pro-inflammatory profile, a process known as ‘inflammaging’. The shift in immune milieu plays a central role in shaping the regenerative capacity of TSCs across multiple organs (Yousefzadeh et al., 2021). These changes are not simply extensions of adult-phase immune remodeling; rather, they signal a new systemic immune landscape with profound implications for stem cell niches. In this context, the Tabula Muris Senis project provides a comprehensive view of immune cell abundance and transcriptional signatures across multiple tissues such as the brain, liver, muscle and skin throughout life. The studies profiled around 350,000 cells across 23 mouse tissues at multiple age points, suggesting intriguing immune signatures. The studies demonstrate that the spleen, one of the main lymphoid tissues, shows a reduction in the proportion of T cells with age, while plasma cell abundance increases. Age-associated perturbation in T-cell populations has been linked to increased prevalence of infections and cancer in the elder population (Almanzar et al., 2020).

**Fig. 1.**
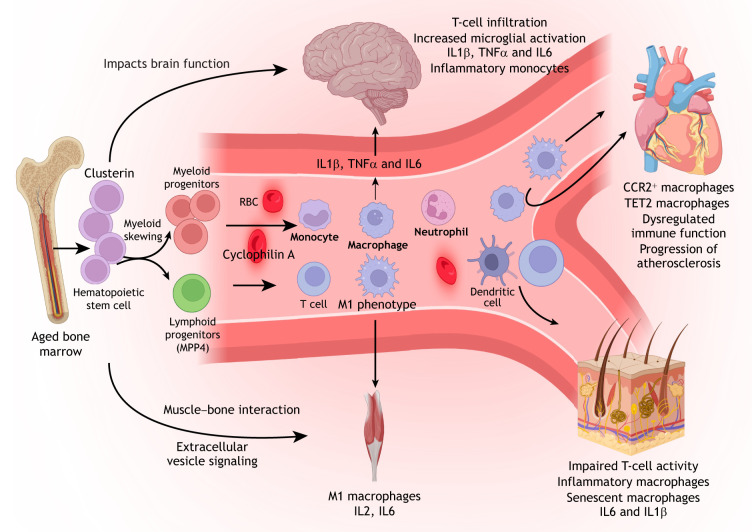
**HSCs as a major driver of systemic aging.** Hematopoietic stem cells (HSCs) preferentially differentiate toward the myeloid lineage, resulting in increased production of monocytes, macrophages and neutrophils, alongside a reduction in lymphoid output. These age-associated shifts not only alter immune cell composition but also promote a pro-inflammatory and anti-healing immune landscape. Inflammatory macrophages adopt an M1 phenotype, release cytokines, and are recruited to peripheral tissues, such as brain, heart and skeletal muscle. Aged brains show increased T-cell infiltration and microglial activation. Clusterin-expressing HSCs contribute to cognitive decline in the brain. Furthermore, aged HSC transplantation promotes the pro-aging factor cyclophilin A in blood and subsequent brain dysfunction. Meanwhile, the aged heart displays accumulation of inflammatory CCR2^+^ and Tet2^lo^ macrophages, predisposing it to chronic inflammation and age-related pathologies, such as atherosclerosis. Aging skin is characterized by inflammatory dendritic cells and dysregulated T-cell responses. RBC, red blood cell. Created in BioRender by Puri, G. 2025. https://BioRender.com/avi8rcc. This figure was sublicensed under CC BY 4.0 terms.

Aged HSCs are thought to be a driver of many of systemic effects, which includes the functional decline of T cells, lineage skewing and altered cytokine profiles that both initiate and regulate niche-level perturbations (Fu et al., 2025). Plasma proteomic studies indicate that aging is not uniform across organs; rather, each organ exhibits distinct aging signatures. Remarkably, approximately 20% of individuals displayed accelerated aging in one organ, while 1.7% showed multi-organ aging (Oh et al., 2023). Aged HSCs may contribute to these systemic aging patterns. As discussed earlier, aged HSCs are biased toward myeloid differentiation and exhibit enhanced secretion of pro-inflammatory cytokines. These circulating factors may mediate inter-organ communication, allowing BM-derived signals to influence the function and regenerative capacity of distant-tissues. Most studies to date emphasize the indirect effects of immune dysfunction on TSCs. Here, we explore how HSC aging throughout the body acts as a principal driver of these immune and regenerative transformations, linking BM niche dysregulation to systemic stem cell function (Fig. 1).

### Nervous system

Microglia, derived from myeloid precursors, are the resident immune cells in the brain, where they maintain a naïve state surveilling for injury or stress signals. Microglia also support the neural stem niche; upon receiving appropriate stimuli, they become activated and release pro/anti-inflammatory cytokines or factors, such as brain derived neurotrophic factor (BDNF) and insulin-like growth factor 1 (IGF1), to maintain homeostasis (Myhre et al., 2019; Gomes et al., 2013). However, as an individual transitions from young adulthood to old age, chronic exposure to inflammatory triggers leads to sustained activation of pro-inflammatory microglia, which impairs their neuroprotective functions and results in reduced neurogenesis in the aged brain (Lucin and Wyss-Coray, 2009; Ximerakis et al., 2019). APPswe/PS1ΔE9 transgenic mice, which exhibit humanized amyloid precursor protein deposition, show increased IGF1 expression in neurons and microglia (Myhre et al., 2019). Aging significantly impacts the central nervous system, impairing cognition and neuronal function, and increasing vulnerability to neurodegenerative diseases. The aged hematopoietic system potentially contributes to this decline by generating dysfunctional immune cells that amplify neuroinflammation, a hallmark of Alzheimer's disease (AD) and Parkinson's disease. This connection is biologically plausible, based on the known effect of aged HSCs on systemic inflammation and immune cell function and has been discussed in previous review articles (Thome et al., 2018; Silvin et al., 2022). Additionally, impaired blood–brain barrier integrity with age permits greater infiltration of peripheral immune cells, such as monocytes and T cells, into the central nervous system, where they accelerate neuronal damage and cognitive decline (Di Benedetto et al., 2017; Shaw et al., 2010; Labzin et al., 2018). As mentioned earlier, the BM microenvironment alters stromal composition, skewing hematopoiesis toward the myeloid lineage. Senescent hematopoietic and stromal cells, along with myeloid-derived monocytes and macrophages, produce proinflammatory cytokines such as IL1β and TNFα. Upon release into the circulation, these factors drive systemic ‘inflammaging’. In the brain, circulating IL1β and TNFα, together with other cytokines, compromise blood–brain barrier integrity, permitting peripheral immune infiltration and exacerbating neuroinflammation (Bourgognon and Cavanagh, 2020; Müller and Di Benedetto, 2024). Clusterin (Clu) is elevated in aged HSCs and has been associated with a shift toward myeloid differentiation (Chambers et al., 2007; Wang et al., 2025b; Koide et al., 2025). Additionally, Clu has been found to be dysregulated in AD pathogenesis. The *CLU* gene contains several AD-associated intronic single-nucleotide polymorphisms, and its protein product is elevated in the brain, cerebrospinal fluid, and plasma of individuals with AD (Lee et al., 2000; Liu et al., 2022) (Table 1). Whether these observations reflect a shared mechanism or represent independent phenomena remains unclear. Another study showed that transplantation of *Clu*-knockout HSCs from aged mice into middle-aged recipients not only restored balanced myeloid output but also improved brain function (Sun et al., 2025). However, the mechanisms by which improved HSC function influences brain function remain to be fully elucidated, and whether Clu itself is directly involved in this process is not yet known. Myeloid skewing may nonetheless represent a major contributor to disrupted brain function in aged mice.

**Table 1.** HSC aging-mediated effects on tissue stem cell function/tissue regeneration

HSC aging alteration	Immune/cytokine change	Impact on tissue/tissue stem cell regeneration	References
Inflammaging	↑ TNFα, ↑ IL1β, ↑ IL6	Amplified neuroinflammation, reduced neural stem cell function	Bourgognon and Cavanagh, 2020; Müller and Di Benedetto, 2024
Myeloid skewing	Expansion of pro-inflammatory myeloid cells	Impaired MuSc regeneration	Dort et al., 2019
Clonal hematopoiesis	Expansion of inflammatory myeloid clones	Promotion of vascular inflammation and worsening of cardiac and muscle stem cell repair	Wong et al., 2021
Senescence and SASP	Inflammatory macrophages	Disrupted wound healing	Dube et al., 2022; Ogata et al., 2021
Pro-aging factors	Cyclophilin A	Cognitive decline	Smith et al., 2020
	Clusterin	Myeloid skewing and effects on brain function	Sun et al., 2025

Published studies reinforce the impact of HSC aging on neural stem cell function, showing that transplantation of aged HSCs into young mice suppresses hippocampal neurogenesis, impairs cognitive function and reduces synaptic density (Smith et al., 2020; Bieri et al., 2023). Conversely, rejuvenating the hematopoietic system by heterochronic BM transplantation with young immune cells improves hippocampal synaptic density, reduces microglial activation and enhances cognitive performance in aged mice (Bieri et al., 2023; Das et al., 2019; Ghahari et al., 2020). Meanwhile, Smith et al. reported that aged HSC transplants elevate levels of the pro-aging factor cyclophilin A (also known as peptidylprolyl isomerase A) in the blood of young recipients, while its inhibition reversed cognitive decline (Smith et al., 2020). Heterochronic parabiosis, an experimental approach that surgically links the circulatory systems of a young and an old animal, has been shown to reverse age-related changes in the aged brain (Villeda et al., 2011). Collectively, these findings emphasize the tight connection between HSC aging and brain function, driving interest in young blood/plasma exchange as a potential therapeutic avenue for neurodegenerative diseases such as AD (Middeldorp et al., 2016; Boada et al., 2020). These findings also exemplify how aging of the hematopoietic system influences distant tissues, reinforcing their role as a key driver of immune dysfunction as well as systemic aging.

### Skeletal system

Numerous studies have demonstrated that muscle and bone represent a similar functional and developmental axis (Ferrucci et al., 2014). Both muscle and bone experience inflammation during aging, with immune cells playing an essential role in maintaining their homeostasis (Joseph et al., 2005; Brotto and Johnson, 2014). Macrophages, among other immune cells, play a central role in coordinating cellular and biological interactions. Muscle stem cells (MuSCs), or satellite cells, are vital for maintaining and repairing skeletal muscle. During homeostatic conditions, MuSCs remain quiescent but become activated upon injury or stress (Pallafacchina et al., 2013). Notably, MuSCs express immune receptors and can directly respond to inflammatory proteins. Age-associated increase of proinflammatory ligands, such as CCR2 ligands and senescence-associated secretory phenotype (SASP) proteins, have been shown to impair MuSC regenerative function directly (Moiseeva et al., 2023; Blanc et al., 2025). Aging diminishes MuSC regenerative capacity through intrinsic defects, such as mitochondrial dysfunction, epigenetic alterations and senescence, as well as extrinsic signals from the local muscle niche and systemic environment. Aged mesenchymal stromal cells also produce fewer trophic factors such as IGF1, impairing MuSC activation and proliferation (Xu et al., 2017).

During muscle regeneration, macrophages not only help to clear the cellular debris upon injury but also produce cytokines, such as TGFβ, to polarize other macrophages towards an anti-inflammatory phenotype. Macrophages can remodel the MuSC niche, providing a pro-regenerative environment through CCR5 (Ratnayake et al., 2021). Additionally, aged BM-derived immune cells adopt proinflammatory phenotypes that disrupt MuSC homeostasis (Tonkin et al., 2015). In line with this, the macrophage pool becomes biased toward a chronic inflammatory M1-like phenotype, impairing the activation and differentiation of satellite cells and leading to defective muscle repair (Dort et al., 2019). TREM2^+^ macrophages have been observed in particular in muscle and bone during aging, a marker of loss of tissue homeostasis. KEGG analysis shows that the transcriptional signature of TREM2^+^ macrophages in muscle is associated with lipid metabolism, lysosomes and phagocytosis. With aging, muscle and bone undergo extensive remodeling characterized by fibrosis, mass loss and microarchitectural deterioration. The emergence of TREM2^+^ macrophages points to a potential driver of these aging-associated pathologies. Additionally, secreted phosphoprotein 1 (SPP1) is a highly expressed marker on TREM2^+^ macrophages. SPP1 has been shown to promote fibrosis in muscular dystrophy and drive bone matrix degradation leading to bone loss, both hallmarks of aging phenotypes (Yin et al., 2024).

Heterochronic parabiosis and BM transplantation models further demonstrate that youthful hematopoietic environments rejuvenate aged MuSCs, whereas aged BM impairs regeneration in young muscles (Rebo et al., 2016; Conboy et al., 2005). Transplantation of young BM cells into old recipients prevents sarcopenia and maintains muscle fiber identity. Conversely, transplanting aged BM into young mice reduces satellite cell numbers and promotes a fibrogenic switch (Wang et al., 2019). Although many studies have confirmed that aged BM impairs MuSC function, it remains unclear whether specific BM lineages, such as HSCs or mesenchymal stromal cells, or niche-derived factors drive this dysfunction. Recent evidence suggests that repopulating the BM with young HSCs improves muscle strength and performance, although the effects on MuSC behavior remain to be fully explored (Wang et al., 2025b).

Importantly, the anatomical proximity between skeletal muscle and BM adds a locoregional component to systemic interactions. Many limb and trunk muscles lie adjacent to active marrow-containing bones, creating a shared microenvironment for paracrine signaling, extracellular vesicle (EV) trafficking and immune cell-derived factor migration (Sui et al., 2024). Therefore, aged BM may exert localized deleterious effects on MuSCs. EVs, including exosomes and micro-vesicles, mediate intercellular communication by transferring regulatory cargo, such as proteins, miRNAs and mRNAs. Although underexplored in this context, a study suggests that EVs may represent an important mode of BM–MuSC interactions (Qin and Dallas, 2019).

Together, these systemic and local interactions position aged BM as a central regulator of muscle regeneration. However, given the logistical challenges of BM transplantation in humans, identifying the specific circulating factors or cell populations responsible for HSC–MuSC crosstalk will be essential for developing practical rejuvenation strategies.

### Skin regeneration

The skin immune system is a complex and indispensable component of the body's defense network, comprising diverse cell types, such as lymphocytes, macrophages, granulocytes, dendritic cells and mast cells, each contributing uniquely to homeostasis and host protection (Zhang et al., 2022; Nestle et al., 2009; Pasparakis et al., 2014). Under steady-state conditions, myeloid cells secrete growth factors that support the survival of keratinocytes, fibroblasts and epithelial cells. During inflammation, these cells rapidly respond by releasing proinflammatory mediators that activate neighboring cells and coordinate the repair response. BM-derived cells are recruited both from the HSC pool, which expands during the early inflammatory phase of wound healing, and from resident myeloid populations that maintain stable representation in the dermis throughout repair (Rodero and Khosrotehrani, 2010). Importantly, skin myeloid cells also bridge innate and adaptive immunity, a function that becomes compromised with age. Immunosenescence leads to the accumulation of dysfunctional macrophages and a reduction in migration ability of Langerhans cells, resulting in impaired repair, disrupted immune communication, and increased susceptibility to infection (Pilkington et al., 2021; Cumberbatch et al., 2002).

In adults, BM-derived HSCs contribute to the early inflammatory phase of wound repair and mesenchymal cells sustain the cell population within the dermis throughout the healing process (Fathke et al., 2004). Importantly, aging HSCs exhibit reduced immune competence, resulting in impaired T-cell, dendritic cell and macrophage function, and therefore disruption of both the inflammatory and tissue remodeling phases of wound repair. In particular, aged macrophages fail to transition efficiently from pro-inflammatory to pro-healing phenotypes, resulting in chronic inflammation and delayed tissue regeneration (Dube et al., 2022). It has been further suggested that aged mice show impeded inflammation resolution and subsequent wound closure due to exaggerated and persistent activation of inflammatory pathways in the macrophages obtained from wound sites (Dube et al., 2022). Although fewer macrophages are found in the aged dermis, they exhibit inflammatory features that are potentially linked to senescence. Cellular senescence has been associated with SASP, characterized by increased inflammatory cytokines and chemokines, proteases and reactive oxygen species resulting in cell death and tissue damage (Ogata et al., 2021; Wilkinson and Hardman, 2020). In another study, it has been confirmed that wound macrophages in diabetic mice with non-healing chronic wounds are associated with CXCR2-dependent SASP (Wilkinson et al., 2019). In homeostatic conditions, IL6 promotes M1-to-M2 polarization of macrophages and as well as extracellular matrix remodeling and re-epithelialization. However, in aged BM IL6 and IL1β are increased, and these factors potentially disrupt IL6 homeostatic function in aged skin (Ho et al., 2019).

Collectively, this evidence highlights how immune dysfunction rooted in HSC aging contributes to impaired skin regeneration and disrupted homeostasis. These findings also suggest a possible link between HSC aging and skin stem cell aging, although further studies are needed to establish this connection.

### Heart regeneration

Cardiovascular disease is one of the leading causes of mortality worldwide, and therefore researchers have great interest in regenerative approaches to treat heart failure. It is well known that the inherent limited regenerative capacity of the heart worsens with age. In post-mitotic tissue such as heart, it remains debatable whether cardiac repair occurs via activation of resident cardiac stem cells and cardiac progenitor cells or through proliferation of pre-existing cardiomyocytes (Mehanna et al., 2022). Fate-mapping mouse models have revealed the immune populations in the heart. Cardiac macrophages, which are negative for the chemokine receptor CCR2, were identified as the major resident cells in the adult heart. These cells originate from embryonic sources, having colonized the heart during development, and sustain themselves in adulthood through local proliferation. CCR2^−^ resident macrophages facilitate angiogenesis and repair (Swirski and Nahrendorf, 2018). Meanwhile, monocyte-derived CCR2^+^ macrophages comprise a minor population in the adult heart, but they can proliferate upon injury or stress and exhibit more pro-inflammatory functions and promote heart dysfunction. Mechanistically, CCR2^−^ macrophages engage with adjacent cardiomyocytes through focal adhesion complexes and respond to mechanical stretch via a TRPV4-dependent pathway, which regulates pro-angiogenesis. These findings highlight the role of tissue-resident macrophages in adaptive cardiac remodeling and identify mechanical sensing as a key trigger of their activation (Wong et al., 2021).

Notably, cardiovascular aging has been linked to genomic instability, telomere shortening and epigenetic alterations, such as DNA methylation and histone modifications. Studies have shown that aged HSCs go through somatic mutations promoting positive selection of certain somatic mutations, giving rise to clonal hematopoiesis of indeterminate potential (CHIP) (further discussed in the ‘Clonal Hematopoiesis’ section) (Jaiswal et al., 2014). CHIP has been strongly associated with cardiovascular aging (Jaiswal and Libby, 2020). In the angiographically controlled Verona Heart Study, 44 older subjects (74-89 years old) with coronary artery disease (CAD) were enrolled. These individuals were then examined for CHIP-associated variants, notably of 11 key genes: *ASXL1*, *DNMT3A*, *IDH1*, *IDH2*, *JAK2*, *PPM1D*, *SF3B1*, *SRSF2*, *TET2*, *TP53* and *U2AF1*. Subjects in the CAD group exhibited a significantly higher variant burden than those in the CAD-free group (72-89 years old), both in terms of the total number of somatic variants and disruptive variants affecting protein function (with significant change in *DNMT3A* and second most effected was *TET2*) (Kwiatkowska et al., 2025). These variants confer a proliferative advantage to specific HSC clones and lead to monocyte skewing and increased production of inflammatory cytokines, such as IL1 and IL6, intensifying in inflammation in CAD (Pardali et al., 2020).

Furthermore, immune senescence in HSC-derived immune cells with aging leads to more complications in cardiac diseases (Zhu et al., 2025). Upon myocardial infarction, neutrophils and macrophages in particular orchestrate post-injury inflammation. Additionally, aging is also associated with functional impairments, consisting of decreased chemotaxis, migration and phagocytic activity as observed in aged neutrophils (Van Avondt et al., 2023). Moreover, due to a lack of anti-inflammatory cytokines, dysregulated neutrophils exacerbate the baseline inflammatory state in response to injury (Butcher et al., 2001). Similarly, macrophages and monocytes display diminished responsiveness to cytokine stimulation, accompanied by production of proinflammatory cytokines. Inflammatory macrophages are also a key player in the development of heart failure with preserved ejection, resulting in remodeling of the left ventricle (Pinto et al., 2014). Macrophage-driven inflammatory factors activate fibroblast and drive extracellular deposition and stiffen the myocardium. This also impairs endothelial-cardiomyocyte crosstalk, worsening diastolic dysfunction of the left ventricle, which is the primary affected site but later involves the right ventricle too due to pulmonary hypertension (Hulsmans et al., 2018). In older individuals, these immune cells show impaired inflammatory resolution, reducing their capacity to support tissue healing and promoting excessive fibrosis and scarring, which attenuates cardiomyocyte regeneration and vascular repair (Sreejit et al., 2020; Wang et al., 2025a). Studies have shown that aged BM-derived myeloid cells exhibit reduced TET2 levels, which is known to epigenetically silence integrin β3, required for cell adhesion and signaling. This suppression activates TNFα signaling, contributing to the conversion of smooth muscle cells into a pro-atherogenic phenotype, and in turn promoting atherosclerotic plaque formation (Kabir et al., 2023). Taken together, aged HSCs contribute to vascular aging and endothelial dysfunction, compounding the regenerative limitations of the heart.

Importantly, studies have shown that rejuvenation of the hematopoietic system can mitigate these deficits. Transplantation of young Sca-1^+^ BM stem cells into aged recipients substantially improves cardiac repair following myocardial infarction. This regenerative benefit was associated with enhanced angiogenic signaling, as reflected by the elevated expression of Cxcl12 and Vegf in donor-derived cells. In parallel, recipient cardiac endothelial cells exhibit increased expression of CXCR4 and phospho-AKT, key mediators of endothelial survival and neovascularization (Li et al., 2019, 2013). These findings underscore the capacity of young HSC transplantation in the heart microenvironment and the role of aged BM in systemic impairment of tissue regeneration.

## Factors mediating HSC-tissue crosstalk

The functional decline of aged HSCs profoundly impacts immune cell production and overall tissue maintenance. These changes reshape the hematopoietic hierarchy and alter the systemic signaling landscape, influencing cytokine profiles, immune cell composition, and inter-organ communication. These shifts create a pro-inflammatory environment capable of impairing the regenerative function of TSCs in distant organs. In the sections below, we explore key mediators of this HSC–TSC crosstalk.

### Immunosenescence

Immunosenescence refers to the gradual decline and remodeling of the immune system with age, marked by reduced responsiveness to pathogens, diminished vaccine efficacy and an increased susceptibility to age-related diseases. This phenomenon involves dysregulation of both the innate and adaptive immune systems (Fu et al., 2025). Immunosenescence is closely associated with aged HSCs, which generate all immune cell lineages. Aging disrupts the self-renewal capacity and cellular function of HSCs, shifting differentiation toward increased myeloid and reduced lymphoid cell production. Recent single-cell RNA sequencing studies of the hematopoietic system, including the BM and spleen, demonstrate significantly decreased production of naïve B and T cells, coupled with expansion of dysfunctional and pro-inflammatory myeloid cells, particularly monocytes and neutrophils (Fu et al., 2025). Recently, a study demonstrated the immune landscape of the hematopoietic immune system among young and aged mice. It was revealed that *Hif1a*, *Cxcr2* and *Cxcl2* expressions were upregulated in old mice particularly. Notably, dysregulated *Hif1a* expression promotes increased neutrophil numbers and activity, further amplifying inflammatory responses, while Cxcr2 and Cxcl2 have a role in immune cell migration and inflammation, respectively (Chaudhary et al., 2025). HIF-α activates NF-κB signaling to regulate neutrophil pro-inflammatory activity and survival under hypoxia, driving prolonged inflammation. The increase in *Hif1a* during aging results in persistent activation of neutrophils and inhibition of neutrophil apoptosis (Lv et al., 2024). As a result, adaptive immunity (e.g. immunological memory) declines, while the innate immune system becomes chronically activated, fueling systemic inflammation (Pritz et al., 2014). Concurrently, aged HSCs accumulate DNA damage, undergo epigenetic drift (progressive alterations in DNA methylation and other epigenetic marks with age), and exhibit a diminished self-renewal capacity. These changes limit immune cell replenishment, contribute to clonal hematopoiesis, and increase the risk of hematologic malignancies (Koide et al., 2021). Collectively, these alterations drive immunosenescence and promote a chronic pro-inflammatory state (inflammaging), which impairs tissue regeneration, accelerates peripheral cellular senescence, and contributes to age-associated diseases. Immunosenescence, therefore, is not merely a reflection of immune system decline, but a systemic consequence of HSC aging on peripheral tissue senescence, impairing regeneration across multiple organ systems.

### Paracrine and systemic signaling factors

Paracrine signaling and immunosenescence synergistically drive age-related decline. Paracrine signaling involves secretion of bioactive molecules, including pro-inflammatory cytokines, growth factors and EVs, by aged BM-derived immune cells. These factors influence both the local BM niche and distant tissues systemically. Recent studies have shown that aged BM-derived monocytes and macrophages drive senescence across multiple organs through the release of EVs, thereby contributing to widespread age-related dysfunction (Hou et al., 2024). This study further examined EVs derived from young and aged BM macrophages (BMMs) and found that miR-378a expression was elevated in senescent BMMs as compared to young controls. One of the downstream targets of miR-378a, peroxisome proliferator-activated receptor α (PPARα), emerged as a potential candidate given its involvement in lipid metabolism, insulin resistance, cardiac aging, inflammation, and renal-associated fibrosis. Pharmacological activation of PPARα using fenofibrate restores tissue homeostasis in aged mice, highlighting its therapeutic potential for mitigating systemic aging and inflammation (Hou et al., 2024).

In parallel, declining levels of IGF1 in the BM during middle age are implicated in HSC aging. Direct stimulation of HSCs with IGF1 has been shown to rejuvenate mitochondrial function and restore the molecular hallmarks of youthful HSCs (Young et al., 2021). Availability of IGF1 also modulates FOXO transcription factors, which are pivotal in maintaining stem cell quiescence. FOXO transcription factors link quiescence to cellular maintenance processes, such as autophagy, integration of systemic signals to regulate inflammation, and stem cell function during aging. Studies have demonstrated that FOXO deficiency disrupts quiescence in HSCs, MuSCs, and neural stem cells (Gopinath et al., 2014; Miyamoto et al., 2007; Renault et al., 2009). As a result, FOXO transcription factors are believed to integrate systemic and paracrine cues to coordinate transcriptional programs that preserve quiescence and regulate inflammation during aging (Audesse et al., 2019; Schäffner et al., 2018).

## Driving mechanisms of HSC aging

Whether intrinsic or extrinsic mechanisms primarily drive aging of HSCs remains a topic of active debate. Parabiosis studies, in which young and old mice share circulatory systems, have demonstrated that young bloodborne factors can restore vasculature, cognitive function, and hematopoietic output in aged mice (Villeda et al., 2014). However, these rejuvenating effects fail to extend to the HSCs themselves (Ho et al., 2021). One limitation of such models is the use of irradiation to prepare recipient mice, which impairs the BM microenvironment and disrupts homing of transplanted HSCs. More recent studies using mobilization-based transplantation strategies have shown partial functional restoration of aged hematopoiesis upon infusion of young donor HSCs (Guderyon et al., 2020). In this section, we summarize the major molecular mechanisms involved in HSC aging.

### Genomic instability

Genomic instability in aged HSCs is characterized by an accumulation of DNA damage, including point mutations, insertions, deletions and chromosomal rearrangements. Aged HSCs exhibit increased replication stress, cell cycle abnormalities and chromosome breaks, which are often indicated by persistent γH2AX signaling (Flach et al., 2014). Reduced expression of mini-chromosome maintenance (MCM) helicases partially drives this instability, impairing ribosome biogenesis and contributing to functional decline. Notably, aged HSCs display a two- to threefold increase in γH2AX foci (Moehrle et al., 2015). To determine the cause of this increase, which revealed that both maintain competent DNA damage response (DDR) activity, as shown by the disappearance of 53BP1 from γH2AX foci following 2 Gy irradiation. Consistently, both groups of HSCs express similar levels of homologous recombination and non-homologous end joining repair genes. However, aged HSCs demonstrate impaired progression through S phase, with stalled or collapsed replication forks activating DDR to restore DNA synthesis. This results in elevated γH2AX levels, indicating that persistent γH2AX in aged HSCs primarily reflects replication stress rather than defective DDR (Flach et al., 2014).

### Epigenetic changes and chromatin accessibility

Epigenetic alterations – heritable changes in gene expression that occur without modifying the DNA sequence – represent another hallmark of HSC aging. In aged HSCs, genes driving myeloid differentiation are upregulated, while those supporting lymphopoiesis become downregulated, reflecting extensive epigenetic reprogramming. Recent studies show that these changes primarily occur in chromatin-accessible regions that are dynamically altered during aging (Itokawa et al., 2022). Although transcriptomic shifts are prominent in downstream progenitors, chromatin accessibility changes are most pronounced in HSCs, diminishing progressively with differentiation. These chromatin regions are enriched in motifs for stress-responsive transcription factors, such as STAT, ATF and CNC families, implicating chronic inflammation and oxidative stress in shaping the aged HSC epigenome (Meng et al., 2023). Many differentially accessible regions align with inactive or primed enhancers, consistent with an ‘epigenetic memory’ or trained immunity model. Although some epigenetic changes may be passive bystanders, others likely actively contribute to dysregulated stress responses and impaired homeostasis in aging HSCs. Unlike transcriptomic profiles, chromatin accessibility and DNA methylation changes remain stable, underscoring the cell-autonomous nature of epigenetic aging (Itokawa et al., 2022).

DNA methylation and histone acetylation are the most studied epigenetic mechanisms that regulate chromatin accessibility and gene expression without altering the DNA sequence. Genomic regions associated with HSC differentiation are hypermethylated in aged HSCs, whereas those linked to self-renewal remain hypomethylated (Sun et al., 2014). DNA methyltransferases (DNMTs) play a central role in this process. For example, DNMT3A and DNMT3B add new methyl groups, whereas DNMT1 preserves methylation patterns during replication. Loss of any of these enzymes disrupts methylation balance, but the functional outcomes differ. Loss of DNMT1 leads to myeloid skewing, whereas DNMT3A deficiency enhances chromatin accessibility at transcriptional sites governing erythroid differentiation (Izzo et al., 2020). Interestingly, epigenetic profiling of aged HSCs has revealed an expansion of H3K27me3-marked regions, a pattern that closely resembles DNMT3A-deficient HSCs (Sun et al., 2014), suggesting that altered DNA methylation is a potential mechanism contributing to HSC aging and the associated perturbations.

Chromatin accessibility and lineage fate are other crucial factors of HSC aging. Lineage bias in HSCs is tightly linked to chromatin accessibility at upstream regulatory elements (UREs). Multipotent HSCs that produce lymphoid cells exhibit open lymphoid-specific UREs, even in the absence of promoter activity, suggesting transcriptionally silent lymphoid priming (Meng et al., 2023). In contrast, platelet-biased HSCs display reduced lymphoid URE accessibility. Aging further reduces chromatin accessibility at lymphoid-specific regulatory regions, impairing lymphoid differentiation. Runx3 overexpression has been shown to restore lymphoid URE accessibility and lymphoid-primed multipotent progenitor (MPP) 4 output in aged platelet-biased HSCs (Meng et al., 2023). Compared to multipotent HSCs, platelet-biased HSCs also generate MPP2 progenitors more rapidly owing to enhanced transcriptional and epigenetic priming of the platelet lineage. Although Runx3 plays a role in lymphoid fate determination, additional factors, including Runx4, are likely involved and merit further investigation.

### Clonal hematopoiesis

Clonal hematopoiesis, which is defined as the expansion of HSC clones harboring somatic mutations, is a hallmark feature of aged hematopoiesis. This process, known as clonal hematopoiesis of indeterminate potential (CHIP), arises from mutations in genes related to epigenetic regulation (e.g. *DNMT3A*, *TET2* and *ASXL1*) (Genovese et al., 2014), RNA splicing (*SF3B1* and *SRSF2*), DDR (*TP53* and *PPM1D*), and signaling pathways (*JAK2* and *CBL*) (Jaiswal et al., 2014; Marnell et al., 2021). These mutations confer selective advantages, such as enhanced self-renewal, impaired differentiation, or resistance to cellular stress, leading to the expansion of mutant clones and reshaping of the hematopoietic landscape with age. Even without overt disease, clonal expansion is associated with increased risks of hematologic malignancies, cardiovascular diseases and other age-related conditions. The selective advantage of these mutant HSCs arises from a combination of intrinsic genetic alterations and extrinsic pressures from the aged BM environment. Loss of function of DNMT3A or TET2 alters methylation programs, promoting self-renewal at the expense of differentiation (Challen et al., 2011; Moran-Crusio et al., 2011). Aging-associated inflammation and genotoxic stress further favor the proliferation of mutant clones. Multiple studies show that IL1 mediates the expansion of *Tet2*^+/−^ hematopoietic stem and progenitor cells (HSPCs) by enhancing their cell cycle progression, repopulating ability and multilineage differentiation over wild-type HSPCs (Caiado et al., 2023; Burns et al., 2022). Furthermore, loss of IL1r1 in *Tet2*^−/−^ HSPCs mitigates key abnormalities of Tet2 deficiency, including increased LSK cells, inflammation and myeloid-lymphoid imbalance. Other inflammatory cytokines, such as IL6 and IFNγ, are also elevated in the aged BM, promoting the expansion of TET2- and DNMT3A-deficient HSCs while suppressing their differentiation (Hormaechea-Agulla et al., 2021). These effects are compounded by autocrine and paracrine feedback loops, wherein mutant HSCs and their myeloid progeny produce additional pro-inflammatory cytokines. Mutations in *TP53* and protein phosphatase 1D confer resistance to chemotherapy and radiation, thereby facilitating clonal survival under cytotoxic stress (Kahn et al., 2018; Hsu et al., 2018; Bondar and Medzhitov, 2010). Together, these factors establish a selective niche in which mutant HSC clones thrive, ultimately compromising hematopoietic functions. These observations underscore a feedback model wherein chronic inflammation not only arises from mutant clones but also reinforces their expansion and functional dominance.

Although these mechanisms have historically been investigated in isolation, accumulating evidence now underscores their profound interdependence. For example, chronic inflammation and replication stress not only fuel genomic instability but also orchestrate epigenetic reprogramming in HSCs, driving clonal selection and skewed lineage commitment (Cao et al., 2024; Avagyan and Zon, 2023; Flach et al., 2014). These observations suggest that what we consider hallmarks of aging are not discrete phenomena occurring independently, but rather components of a complex, integrated network of cellular and systemic events that collectively propel the aging process. Recognizing and unraveling these intricate connections is essential for a holistic understanding of organismal aging and for the development of targeted interventions.

## Therapeutic implications and potential strategies

Aging induces both intrinsic defects within HSCs and extrinsic alterations in the BM niche, increasing the risk of hematologic malignancies frequently treated by hematopoietic stem cell transplantation. Over the past two decades, the frequency of hematopoietic stem cell transplantation has substantially increased among older adults. However, older transplant recipients often experience heightened age-related complications compared to the general population. Clinically, it has been suggested that the physiological stress and associated therapies of transplantation may accelerate biological aging (Cupit-Link et al., 2018).

**Fig. 2.**
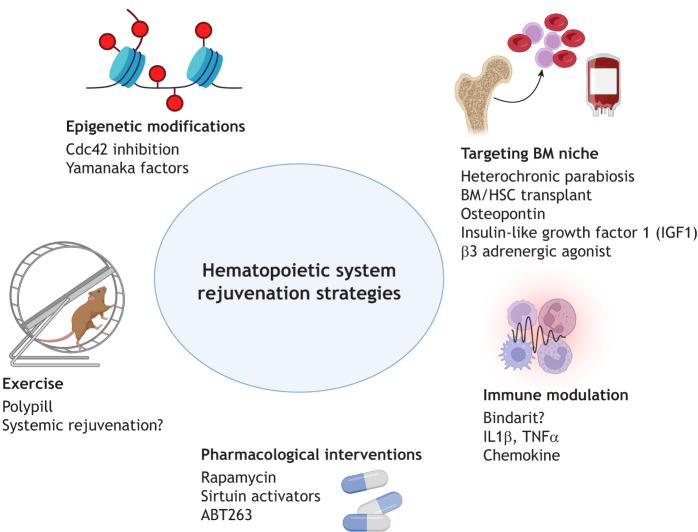
**Strategies to rejuvenate the hematopoietic system.** Multiple approaches have been explored to restore hematopoietic stem cell (HSC) function and bone marrow (BM) niche integrity during aging. Heterochronic plasma transfer (parabiosis) from young counterparts is a well-known strategy to improve HSC function in aged recipients. Niche-target factors, such as osteopontin and insulin-like growth factor 1 (IGF1), support regeneration of the BM microenvironment. Epigenetic reprogramming, through modulation of chromatin accessibility and transcriptional resetting, offers another avenue for rejuvenation. Immune modulation, including cytokine inhibition and anti-inflammatory therapies, aim to mitigate the chronic inflammatory milieu associated with aging. Lifestyle intervention, such as exercise, has emerged as a potent systemic rejuvenator. Pharmacological targets, such as mTOR inhibition with rapamycin and senolytic agents such as ABT263 targeting metabolic and apoptotic pathways, offer additional therapeutic avenues. Although several of these strategies show encouraging results, many require validation in preclinical and clinical settings. In most instances, the potential systemic effects of hematopoietic rejuvenation therapies has not been directly investigated. Created in BioRender by Puri, G. 2025. https://BioRender.com/cxm9c3c. This figure was sublicensed under CC BY 4.0 terms.

Reduced-intensity conditioning regimens were developed to mitigate adverse outcomes, effectively reducing transplant-related mortality, but they carry a higher risk of relapse. Conversely, high-intensity regimens lower relapse risks but elevate transplant-related mortality, particularly among older individuals. These poor outcomes largely arise from age-related impairments in hematopoietic regeneration and functional decline within the BM niche (Ramalingam et al., 2023). Considering the pivotal role of HSCs and the BM niche in both tissue regeneration during aging and transplant success, there is an urgent need to develop therapeutic strategies that rejuvenate these components to sustain lifelong hematopoietic function (Fig. 2).

Emerging therapeutic strategies to rejuvenate hematopoiesis increasingly target both intrinsic cellular mechanisms and extrinsic niche environments. For example, niche-focused interventions aim to restore supportive structural and cellular components of the aged BM microenvironment. Promising approaches include transplantation of young BM endothelial cells, treatment with β3-adrenergic agonists (Ho et al., 2019), recombinant osteopontin (Guidi et al., 2017) and IGF1 (Young et al., 2021), each demonstrating efficacy in restoring aged HSC function. Myeloid-lymphoid imbalance is a well-studied hallmark of immune aging and a potential target for rejuvenation strategies. A recent study conducted by Ross et al. found that depletion of myeloid-biased HSCs in aged mice resulted in a balanced HSC pool and restored immune environment. This was accompanied by an increase in lymphoid progenitors and naïve cells and reduction of lymphocyte exhaustion. Notably, aged mice with a rebalanced HSC pool exhibited protective immunity against live, pathogenic retroviral infection. Although targeting myeloid-biased HSCs is a promising approach for human immune rejuvenation, further studies are needed to develop safe antibody-based strategies and to ensure that differentiated cells, such as regulatory T cells, are not unintentionally affected (Ross et al., 2024). Another study showed that modulation of the pro-inflammatory BM milieu, particularly by blocking cytokines such as IL1, has been effective in reversing myeloid bias and improving hematopoietic regeneration (Kovtonyuk et al., 2022). Although IL1 inhibition has not yet been extensively evaluated in the context of MuSC aging, its relevance is underscored by the roles of inflammatory cytokines and chemokines in MuSC dysfunction and repair. Our recently published data suggest that bindarit, a monocyte chemoattractant inhibitor, ameliorates systemic inflammatory profiles, reflected by balanced blood populations (neutrophils, monocyte, lymphocytes) and inflammatory cytokines (TNFα and IL6) in old mice. The benefit of bindarit also extends to enhancement of muscle regeneration and strength (Blanc et al., 2025). These findings raise the possibility that bindarit may modulate the hematopoietic system to exert its effects on peripheral tissues. However, further investigations are needed to elucidate the underlying mechanism(s).

Epigenetic rejuvenation strategies aim to reset age-associated transcriptional and chromatin landscapes. Cdc42, a member of the Rho GTPase family, has an active GTP-bound and an inactive GDP-bound state. It is a key regulator of cytoskeletal dynamics, controlling actin and tubulin organization, cell–cell and cell–extracellular matrix adhesion, as well as cell polarity across diverse cell types. Pharmacological inhibition of Cdc42 activity has been shown to functionally rejuvenate aged HSCs, enhance the proportion of polarized cells within the aged HSC pool, and restore both the levels and spatial distribution of histone H4 lysine 16 acetylation to a state comparable to that of young HSCs (Florian et al., 2012). Another strategy involves epigenetic reprogramming through the overexpression of Yamanaka factors [Oct4 (Pou5f1), Sox2, Klf4, and Myc], which can shift aged cells and tissues toward a more embryonic-like state (Kasbekar et al., 2023). Induced pluripotent stem cell reprogramming of HSCs from 23-month-old mice, followed by redifferentiation into HSCs (iHSCs), enabled T-cell chimerism upon transplantation. Notably, iHSCs derived from aged HSC clones that previously lacked T-cell potential regained the capacity to generate T cells (Wahlestedt et al., 2017; Kasbekar et al., 2023). Despite the promise of these strategies, significant challenges remain, notably the potential risk of tumorigenesis and concerns about maintaining lineage fidelity. Further studies are needed to determine whether *in vivo* induction of Yamanaka factors can delay hematopoietic aging or rejuvenate the function of aged HSCs.

Targeting metabolic dysregulation also offers therapeutic promise. Agents such as rapamycin (mTOR inhibitor), sirtuin activators, and NAD+ precursors (e.g. nicotinamide riboside) have successfully restored mitochondrial fitness and enhanced engraftment potential in aged HSCs (Chen et al., 2009; Chiang et al., 2025). Additionally, senolytic approaches aiming to selectively eliminate dysfunctional HSCs represent another promising therapeutic avenue. Navitoclax (ABT263) is one of the known potent senolytic agents, effectively targeting senescent cells irrespective of the pathway by which senescence is induced. Administration of ABT263 has been shown to clear irradiation-induced senescent cells and restore functional capacity of HSCs (Chang et al., 2016). Furthermore, immune-based approaches leveraging neoantigen recognition or natural killer cell-mediated killing via NKG2D ligands have been explored (Paczulla et al., 2019). However, achieving specificity without off-target toxicity remains a key challenge.

Apart from direct cellular and molecular interventions within the BM, there is growing interest in plasma-based interventions that aim to alter the systemic environment driving age-related decline. Multiple studies have shown that exposure to young blood can reverse aspects of aging in various organs in mice (Villeda et al., 2014; Rebo et al., 2016). However, its clinical relevance in humans remains uncertain. This approach is also financially burdensome; each plasma infusion requires multiple donors and logical scalability is limited. More recent research has shifted focus from supplementing young blood to depletion of pro-aging factors, which may offer a more practical and mechanistically sound therapeutic approach (Mehdipour et al., 2020; Rebo et al., 2016). To advance this field, further large-scale, multidisciplinary studies are required to identify and examine functional relevance of niche-specific and systemic pro-aging factors in humans that could be targeted to rejuvenate HSCs and the BM microenvironment.

A different approach includes exercise (also known as polypill to treat/prevent chronic diseases; Pareja-Galeano et al., 2015) or lifestyle changes, which have been shown to influence the hematopoietic system, particularly by modulating activated hematopoietic progenitors cells residing within the vascular niche (Baker et al., 2011). A recent study has explored the beneficial potential of volunteer wheel running on aged stem cells, including HSCs and HSPCs. Studies have revealed that exercise mitigates the heightened inflammatory landscape in aged HSPCs (Liu et al., 2023a). Although the underlying mechanisms were not directly addressed in this study, restoration of quiescence likely represents a plausible explanation. Another study reported an increase in lymphoid cell numbers following exercise, without a corresponding rise in HSC frequency (Shen et al., 2021), suggesting a shift in lineage output rather than stem cell expansion. Despite the promising findings, several important questions remain unanswered regarding the sustainability of exercise-induced benefits on aged HSCs. Additionally, it is still unclear which systemic or niche-derived factors mediate the benefits of exercise, and whether these outcomes depend on the type, intensity or duration of physical activity.

Collectively, these therapeutic strategies reflect the multifactorial nature of HSC aging and highlight complementary approaches currently under investigation to extend the hematopoietic healthspan and lifespan.

## Conclusions and future outlooks

As outlined in this Review, the decline in HSC function with age has profound consequences extending beyond the hematopoietic system. It is increasingly recognized that aging represents a systemic, multi-organ process orchestrated by intricate intracellular and inter-organ signaling networks. This complexity poses a significant challenge for reductionist approaches to studying aging. Aged HSCs produce dysfunctional immune cells, secrete inflammatory mediators, and exhibit altered lineage differentiation, collectively influencing tissue health throughout the body (Fulop et al., 2023; Fotopoulou et al., 2025). Additionally, organs such as the liver, spleen, and gut transmit feedback signals to the hematopoietic system via metabolic, inflammatory and hormonal pathways (Wells et al., 2024). Disruption of these multidirectional and nonlinear interactions contribute significantly to organism-wide tissue degeneration and impaired regeneration during aging.

To address this challenge, researchers in regenerative biology are increasingly utilizing AI-assisted computational methods and systems biology approaches (Zhavoronkov et al., 2019). Emerging tools such as deep learning, graph-based modeling, and multi-omics integration enable simulation of complex organ-to-organ interactions and prediction of systemic outcomes following age-related perturbations. These computational approaches facilitate comprehensive and unbiased integration of transcriptomic, proteomic, metabolomic, cytokine and epigenomic data across diverse cell types and distant tissues (Ahadi et al., 2020; Menche et al., 2015). For instance, network modeling has uncovered central nodes of inflammation and immune dysfunction that potentially stem from hematopoietic dysregulation (Ahadi et al., 2020). Such models have identified predictive biomarkers and have suggested therapeutic targets with the potential to delay or reverse age-related dysfunction across multiple tissues. Notably, most existing studies highlight the indirect effects of HSC aging or immune dysfunction on tissue aging. However, the direct impact of HSC or immune dysfunction on TSCs remains an important area for further investigation.

This emerging model positions HSC aging as a central driver of systemic inflammation, leading to disruption of tissue-specific stem cell niches and accelerating organismal aging. This framework reframes the hematopoietic system as a strategic therapeutic target not only for hematologic and immune disorders but also for chronic degenerative conditions affecting muscle, skin, brain and heart. Thus, the aforementioned interventions targeting HSC dysfunction represent a compelling strategy to address aging at its systemic root. Although several methods have demonstrated promise in preclinical models, clinical translation will require optimization of delivery systems, comprehensive long-term safety validation, and integration within broader systemic aging interventions. Moreover, identifying therapies capable of reversing or delaying systemic aging – beyond localized stem cell rejuvenation – remains an important and unmet goal. This goal has never felt more approachable than today thanks to the rise of powerful computational technology, and the growing efforts to initiate multidisciplinary large-scale projects within the scientific community.

Ultimately, by addressing hematopoietic aging through these multifaceted approaches, the scientific community moves closer to effectively counteracting systemic aging, promoting healthier aging, and significantly enhancing the quality of life in older adults.
